# Gastrointestinal Microbiota and Type 1 Diabetes Mellitus: The State of Art

**DOI:** 10.3390/jcm8111843

**Published:** 2019-11-02

**Authors:** Marilena Durazzo, Arianna Ferro, Gabriella Gruden

**Affiliations:** Department of Medical Sciences, University of Turin, C.so A.M. Dogliotti 14, 10126 Turin, Italy; arianna.ferro@unito.it (A.F.); gabriella.gruden@unito.it (G.G.)

**Keywords:** type 1 diabetes, microbiota, microbiome, auto-immunity, gut permeability

## Abstract

The incidence of autoimmune type 1 diabetes (T1DM) is increasing worldwide and disease onset tends to occur at a younger age. Unfortunately, clinical trials aiming to detect predictive factors of disease, in individuals with a high risk of T1DM, reported negative results. Hence, actually there are no tools or strategies to prevent T1DM onset. The importance of the gut microbiome in autoimmune diseases is increasingly recognized and recent data suggest that intestinal dysbiosis has a pathogenic role in T1DM by affecting both intestinal immunostasis and the permeability of the gut barrier. An improved understanding of the mechanisms whereby dysbiosis in the gut favors T1DM development may help develop new intervention strategies to reduce both the incidence and burden of T1DM. This review summarizes available data on the associations between gut microbiota and T1DM in both experimental animals and humans and discusses future perspectives in this novel and exciting area of research.

## 1. Introduction

In the last decade, the gut microbiota has gained increasing interest because of a major shift in our way of thinking: the microbiota is no longer considered a passive collection of microbes hosted in the gut, but an integral part of the body, providing important functions to the host. Landmark studies have significantly improved our understanding of microbiota function, dynamic, and interactions. Today, we know that the gut microbiota is involved in both the development and progression of many pathological conditions and targeting the microbiota has become a novel promising intervention strategy. Recent data suggest that an imbalance in gut microbiota (dysbiosis) could be involved in the pathogenesis of autoimmune type 1 diabetes (T1DM) and may contribute to explaining the global rise in T1DM incidence, particularly in childhood [1,2,3]. Indeed, a dysfunctional microbiota can favor the development of autoimmune diseases by altering the maturation of the immune system in early infancy and by increasing both intestinal permeability and inflammation. Despite major research effort, clinical trials in individuals with high T1DM risk reported negative results and there is no effective strategy for the primary prevention of T1DM. Hopefully, studying the role of the intestinal microbiota in T1DM may provide insights into developing new strategies to reduce T1DM incidence. 

## 2. Type 1 Diabetes Mellitus

T1DM accounts for 5–10% of all diagnosed cases of diabetes and onset of the disease usually occurs during childhood or adolescence [4]. The International Diabetes Federation (IDF) estimated that more than a million subjects (<20 years) were affected by T1DM in 2017 and approximately 86,000 children develop T1DM every year; however, the incidence varies among countries and even in different areas within the same country [5]. In Europe, there is a north–south gradient, with Scandinavian countries exhibiting the highest incidence rate. The incidence is much lower in the Mediterranean area, though Sardinia has the second highest incidence in the world after Finland [6]. T1DM incidence has increased over the last 50–60 years in many countries, with an approximate 3% rise in incidence rate per year [6,7]. In some countries, the disease is also occurring at a much earlier age, with a marked increase in the 0–4 years age range [8], indicating a lowered threshold for T1DM development.

T1DM is characterized by absolute insulin deficiency due to pancreatic β cell loss. This process, occurring in genetically susceptible individuals, may be triggered by environmental factors, and progresses over many months or years, during which the subject is asymptomatic and euglycemic. Indeed, autoimmune β cell destruction by autoimmune T cells progresses slowly and hyperglycemia and symptomatic diabetes only develops when a critical mass of β cells (~80%) is lost. Therefore, clinical T1DM is preceded by a silent phase in which the presence of autoantibodies (insulin, glutamic acid decarboxylase, insulinoma-2-associated, and zinc transporter 8 autoantibodies), which are biomarkers of T1DM-associated autoimmunity, is the only detectable abnormality [9,10]. 

Clinical onset is characterized by polyuria, thirst, polydipsia, weight loss, hunger, polyphagia, and fatigue. Early recognition is important to prevent the development of diabetic ketoacidosis (DKA), a severe condition characterized by hyperglycemia, acidosis, ketonemia, and ketonuria. DKA occurs at the time of T1DM diagnosis in approximately 30% of children in the United States and the incidence rate in children with established diabetes is approximately 6–8% per year. DKA is the leading cause of morbidity and mortality in pediatric T1DM. Indeed, in children, cerebral injury occurs in 0.3 to 0.9 percent of DKA episodes and has a mortality of 20–25% [11]. 

In the absence of DKA, T1DM diagnosis is based on one of the following criteria: (1) random plasma glucose ≥ 200 mg/dL in a patient with classic symptoms of hyperglycemia, (2) fasting plasma glucose ≥ 126 mg/dL, (3) plasma glucose ≥ 200 mg/dL two hours after a glucose load of 1.75 g/kg (oGTT), and (4) glycated hemoglobin (HbA1C) ≥ 6.5%. Unless symptomatic hyperglycemia is present, the diagnosis should be confirmed by repeat testing [4]. Because of the rapid T1DM onset, HbA1C may not be markedly elevated; therefore, an HbA1C level below 6.5% does not exclude the diagnosis of diabetes in pediatric patients.

Once it is diagnosed, T1DM requires immediate treatment with insulin replacement therapy. Intensive insulin therapy combines the administration of long-acting basal insulin together with pre-meal boluses of rapid-acting insulin to mimic the physiological secretion of insulin by the β cells. This can be achieved by either Multiple Daily insulin Injections (MDI) or Continuous Subcutaneous Insulin Infusion (CSII). The general aim of T1DM therapy is to maintain glucose control as near to normal as possible without causing hypoglycemia. In addition, T1DM care should also include diabetes education, self-management training, patient-tailored plans on healthy nutrition and exercise, and psychosocial support [4].

To prevent the burden of T1DM care, the best approach would be the primary prevention of T1DM in subjects at risk of developing the disease [12]. However, clinical trials focusing on primary prevention failed to prove benefit so far and there is no effective tool to block the development of the disease [12]. This is, at least in part, due to our poor understanding of T1DM pathogenesis. *HLA-DR* and -*DQ* genes account for 40–50% of the susceptibility to the disease [13]. Moreover, recent studies suggest an important role of gene polymorphisms, such as Toll-like receptor exon polymorphisms and polymorphisms in genes critical for vitamin D metabolism [14,15,16,17]. However, genetics alone cannot explain T1DM onset, as less than 10% of the children, who are genetically predisposed, develop the disease. Thus, environmental factors that affect the immune response, changing the probability of an autoimmune reaction against β cells in genetically susceptible subjects, play a crucial role. Environmental influences implicated in the pathogenesis of T1DM include pregnancy-related and perinatal factors, viruses, ingestion of cow’s milk and cereals, and use of both antibiotics and probiotics [18]. These factors also affect the microbiota, adding to the hypothesis of a role of the gut microbiome in linking environment influences with the development of T1DM.

## 3. The Microbiota

The microbiota is defined as the set of microorganisms living in symbiosis with their human/animal host [19]. The human microbiota consists of symbiotic microbial cells harbored on skin, respiratory and urogenital systems and gastrointestinal tract. In the latter, microbial populations reach the highest density and form a complex microbial community of ~4 × 10^13^ cells [19,20]. Although the gut microbiota includes different types of microorganisms, bacteria are the best studied. Sequencing the 16S rRNA is a key methodology to identify and classify these bacterial populations [21,22]. Furthermore, new metagenomic methods can precisely identify functional and strain-specific differences in the microbiome [23]. Healthy adults share most gut bacterial populations, which constitute a “core microbiota”. In fact, even if thousands of bacterial species have been isolated from human intestine, most of them belong to just four phyla: Bacteroidetes and Firmicutes are predominant (90%), while Actinobacteria and Proteobacteria are less abundant [24]. However, the concept of the “core microbiota” has been challenged by data showing a vast microbial diversity both over time and across human populations [25]. 

Colonization of the gut starts at birth, though recent studies questioned the dogma that the womb is a sterile environment, and is markedly affected by the mode of delivery. Vaginally delivered infants acquire microbial communities resembling maternal vaginal microbiota, predominantly Lactobacillus. In contrast, C-section infants harbor bacterial strains similar to those found on the skin, such as Staphylococcus and Clostridium genera [26,27]. Fecal microbiota resembles that of the mothers in 72% of vaginally delivered infants, while this percentage is reduced to 41% in babies delivered by C-section [26]. However, recent evidence shows that maternal skin and vaginal strains only transiently colonize the infant and that mother-to-infant transmission of gut strains, which occurs after birth via unclear mechanisms, is more relevant and persistent [28].

The intestinal microbiota of newborns is generally low in diversity and is dominated by two main phyla: Actinobacteria and Proteobacteria. Later, the gut microbiota becomes more diverse, with the predominance of Firmicutes and Bacteroidetes. By approximately 2–3 years of age, the composition, diversity and function of the microbiota are very similar to that of adult subjects. 

Although the gut microbiota is relatively stable in adults compared to infants, it can undergo major changes in response to environmental influences, such as diet composition, the use of drugs, the lifestyle, environment hygiene standards and geographical location [29]. For instance, diet composition can modulate gut bacteria composition since childhood [26,30]. In breastfed infants, the gut microbiota is characterized by higher levels of probiotic genera, such as Lactobacillus and Bifidobacterium, and shows a lower microbial diversity compared to that of formula-fed babies [26,30]. Moreover, the consumption of animal proteins is positively associated with the presence of Bacteroides and Alistipes genera, while fiber intake is linked to increased Bifidobacterium and Lactobacillium genera, both in childhood and adulthood [31,32]. These genera are also enhanced by supplementation with probiotics [31,33]. Other environmental factors that greatly influence the gut microbiota composition are the use of drugs, particularly antibiotics, and geographical location [25]. Although it is well established that antibiotic therapy can reshape the gut microbiota, it remains unclear whether the microbial community can fully recover following antibiotic treatment [34,35]. 

The gut microbiota becomes unstable and less diverse with aging and the centenarian microbiota shows an increase in facultative anaerobe species, such as Escherichia coli, and a rearrangement of the profile of butyrate producers, such as a decrease in Faecalibacterium prausnitzii [36]. These events are closely associated with both an age-related decline in immunocompetence and coexisting illnesses [36]. 

## 4. Importance of the Microbiota to the Host

The human gut microbiota plays a multitude of important functions. It provides protection against pathogen overgrowth, preventing their colonization via the inhibition of adherence, production of bacteriocins, and both site and nutrient competition. It metabolizes specific drugs and eliminates exogenous toxins, thus playing an important detoxifying role. It is involved in the synthesis of essential vitamins, such as B1, B2, B5, B6, B12, K, folic acid, and performs biliary acid deconjugation. In addition, fermentation of indigestible carbohydrates by intestinal bacteria allows their use as a source of energy and provides 5–10% of daily energy requirement [37,38]. Furthermore, in the normal colon, the gut microbiota contributes to intestinal repair by promoting cellular proliferation and differentiation and it is of paramount importance in maintaining the integrity of the gut barrier [37]. Growing evidence points to a key role of the gut microbiota in both maturation and continued education of the host immune system [39]. Indeed, the immune system learns to discriminate between commensal and pathogenic bacteria and, following education, commensal bacteria triggers an anti-inflammatory response, while pathogenic bacteria a pro-inflammatory reaction. Moreover, the gut microbiota modulates both the migration and function of neutrophils and affects T cell differentiation, favoring both the differentiation and expansion of regulatory T cells (Tregs), which are key mediators of immune tolerance. An important mechanism by which the gut microbiota controls the immune system is through the formation of short-chain fatty acids (SCFAs), such as butyrate, acetate, and propionate [38,40]. SCFAs are mainly generated by bacterial fermentation of non-digestible carbohydrates, such as dietary fiber [40]. In T lymphocytes, SCFAs activate G protein-coupled receptors (GPR41/GPR43) signaling pathways, inhibit histone deacetylases, and induce metabolic alterations by enhancing the activity of the mammalian target of rapamycin (mTOR) complex. This results in the inhibition of inflammatory cascades, the expansion of mucosal Tregs, and the decreased production of inflammatory cytokines, such as interleukin-10 (IL-10) and interferon-γ (IFN-γ) [40]. 

Given the key role of the gut microbiota in maintaining the host health, it is not surprising that alterations in the microbiota (dysbiosis) could be implicated in the pathogenesis of a growing number of extraintestinal diseases including T1DM [41,42].

The term “dysbiosis” refers to an imbalance of the gut microbiota due to the loss of beneficial microbial organisms, the expansion of potentially harmful microorganisms and/or lower microbial diversity [43,44]. Although this term has been widely used in the field of microbiota research, the concept of gut microbiota dysbiosis, which is predominantly based on early microbiome taxonomic studies, is poorly defined and has been recently questioned [45]. Given the complexity and great variability of the human microbiota, changes in the microbiota may be irrelevant to the host health and there is an increasing quest for more rigorous criteria, such as modified Koch criteria, to identify abnormalities pertinent to the microbiota that can be of clinical relevance.

## 5. Microbiota and Type 1 Diabetes Mellitus

### 5.1. The Microbiota in Experimental T1DM

The possible role of the gut microbiota in the pathogenesis of autoimmune diabetes was first suggested in the 1980s, but scientific interest on this topic has dramatically increased in the last two decades. Experimental studies have been predominantly performed in animal models of autoimmune diabetes such as Non-Obese Diabetic (NOD) mice and BioBreeding diabetes-prone rats. Consistent with the hypothesis that dysbiosis occurs in T1DM, most of the studies in experimental autoimmune diabetes reported changes in both diversity and composition of gut microbiota. Moreover, fecal transplantation from NOD to Non-Obese Diabetes-Resistant (NOR) mice caused insulitis, indicating a diabetogenic effect of the NOD microbiota [46]. Cross fostering is another effective mean to induce a sustained microbial shift. Cross fostering of NOD mice by NOR mice was capable to protect from diabetes. Specifically, no development of diabetes was observed in NOD male mice nursed by a NOR mother, compared to an 80% incidence of T1DM in male NOD mice nursed by NOD mothers [47].

The link between microbiota-induced innate immunity alterations and T1DM risk was highlighted by studies performed in animals lacking Myeloid differentiation primary response 88 (MyD88), an adaptor protein for the toll-like receptors that recognize microbial patterns and regulate the innate immune response [48]. NOD mice lacking MyD88 were protected from T1DM, indicating that MyD88 is important in T1DM development [48]. However, MyD88 deletion in germ-free NOD mice increased the risk of T1DM [48]. Therefore, the protective effect of MyD88 deficiency is microbiota-dependent, confirming that interactions between the microbiota and innate immunity are important in the development of the disease. In addition, inflammatory lymphocytes, such as T helper 17 and type 3 innate lymphoid cells, are increased in the intestinal lamina propria of NOD mice, while tolerogenic dendritic cells and Treg are reduced in the lymph nodes draining the gut [49]. These changes in adaptive immunity may favor the activation of auto-reactive T cells against β cell antigens and thus T1DM development.

Dysbiosis can also affect the integrity of the gut barrier, resulting in enhanced permeability and the transit of antigens. Tight junctions, sealing the gap between adjacent intestinal epithelial cells, control gut permeability, allowing nutrient absorption, while preventing the transit of dietary, bacterial, and viral antigens. The mucus layer covering the epithelium represents another important barrier and controls the type of commensal bacteria residing in the epithelium [50]. In early experimental autoimmune diabetes prior to the development of insulitis, dysbiosis and altered immunostasis are paralleled by abnormalities of the gut barrier, such as decreased numbers of goblet cells and diminished mucus production [49], leading to enhanced intestinal permeability [51,52,53]. Cross fostering of NOD mice by NOR mothers corrected the defect in mucus production, indicating a key role of NOD microbiota in the dysfunction of the gut barrier [49]. 

How the loss of gut barrier integrity triggers β cell autoimmunity remains unclear. It has been proposed that the entrance of antigenic bacterial components into the systemic circulation can directly induce β cell damage and/or activate β cell autoimmunity in the pancreatic lymph nodes. Consistent with this hypothesis, translocation of the microbiota to the pancreatic lymph nodes has been observed in the NOD model [49]. Alternatively, T cells recognizing β cell antigens can be activated by bacterial products in the gut and then travel to the pancreatic lymph nodes to induce β cell injury [54]. In line with this, the induction of chronic colitis, which alters both gut barrier integrity and mucus layer composition, triggered the activation of islet-reactive T cells in the gut mucosa and led to autoimmune diabetes in BDC2.5XNOD mice, an animal model mimicking subjects at high risk of T1DM. Moreover, treatment of these mice with antibiotics prevented T1DM, confirming that the microbiota was required to induce β cell autoimmunity [55]

Taken together, results from experimental animals support the hypothesis that the microbiota may play a role in the pathogenesis of T1DM by affecting both the immune response and the gut barrier integrity. However, the mechanisms leading to β cell autoimmunity are still unclear and require further investigations. 

### 5.2. Intervention Studies in Experimental Autoimmune Diabetes 

Several studies in susceptible experimental animals have explored whether intervention strategies affecting the microbiota can modulate the risk of developing T1DM. Probiotic supplementation [56,57,58], prebiotic assumption [59], antibiotic therapy [46,60,61,62,63,64], and SCFA supplementation [65,66] has been shown to reduce the risk of T1DM onset. NOD pups born to mothers treated with broad-spectrum antibiotics showed increased T1DM incidence together with changes in the microbiota and inflammatory alterations in the intestinal mucosa [67]. Similarly, NOD mice intermittently treated with a macrolide in early life showed increased insulitis, enhanced T1DM incidence, and abnormalities in both innate immunity and T cell differentiation [63]. More recently, a study has shown that a single early-life antibiotic course accelerated T1DM development in male NOD mice and this was paralleled by persistent changes in the gut microbiome and altered the expression of genes controlling both innate and adaptive immunity [68]. On the contrary, supplementation with VSL#3, a probiotic preparation containing eight different strains, decreased T1DM incidence in NOD mice [56]. Similarly, high doses of butyrate in the diet significantly reduced T1DM incidence in NOD mice through the expansion of Treg cells [65]. Recently, supplementation with low-methoxyl pectin (LMP) dietary fiber has been shown to reduce T1DM incidence and fasting glucose levels in NOD mice. This was paralleled by the enrichment of bacterial species producing SCFAs, reduced inflammation, the amelioration of gut barrier integrity and an increase in Treg cells. Antibiotic treatment worsened T1DM autoimmunity in this model, but the transfer of feces from LMP-fed mice reversed the effect of antibiotics [69], confirming the key role of the microbiome in mediating the beneficial effects of LMP. Treatment of NOD mice with the probiotic bacteria Clostridium butyricum increased Tregs in both mesenteric and pancreatic lymph nodes, enhanced expression of microbial genes important in butyrate production, and delayed the onset of T1DM [70]. 

**Figure 1:**
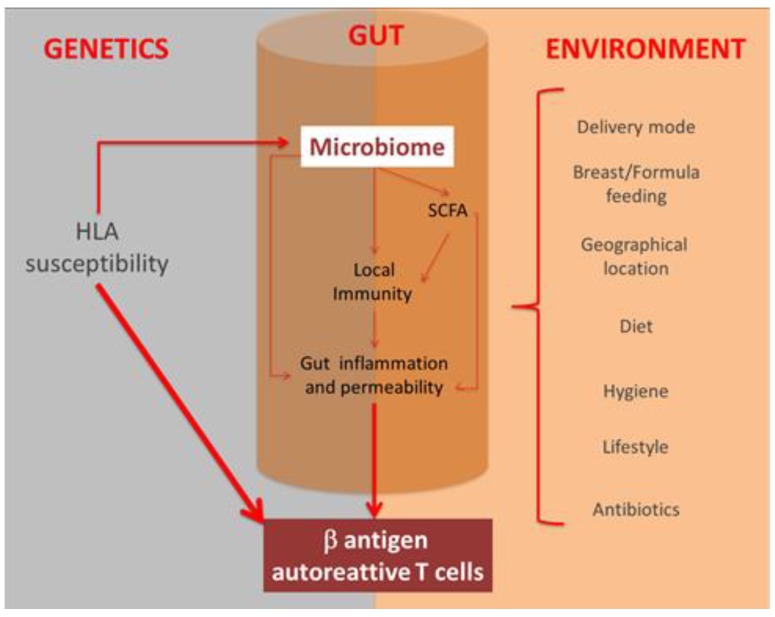
Interplay between genetics, environment, and gut microbiota in T1DM pathogenesis. Specific *HLA* allele can increase susceptibility to the development of type 1 diabetes and influence the composition of the gut microbiota. Many environmental factors can also shape the gut microbiota. Abnormalities in the gut microbiome are believed to favor autoimmunity against pancreatic b-cells by modulating the gut immune/inflammatory response and by enhancing the gut barrier permeability both directly and indirectly through reduced production of short-chain fatty acids (SCFAs).

Taken together, these findings support the hypothesis of a causal link between changes in the microbiota composition and the risk of T1DM onset and they also suggest potential therapeutic strategies to restore a healthy microbiome. However, inbred mouse strains are genetically homogenous, and thus they cannot capture the inherent genetic variations of humans. Moreover, a number of factors (i.e., mode of delivery, diet, social activity) that shape human microbiota are absent in mice. Therefore, results from animal models are not easily translatable to humans and conclusions in experimental models should be taken with caution (Figure 1).

### 5.3. The Microbiota in Human T1DM

Table 1 summarizes both the design and results of a selection of recent studies performed in humans [1]. Studies exploring the role of the microbiota in human T1DM have been performed in different stages of the disease, ranging from genetic predisposition to clinical diabetes [71,72,73,74,75,76,77,78,79,80,81,82]. To support the hypothesis of a microbiota contribution to T1DM pathogenesis, studies carried out in the preclinical stage of the disease, identified by the appearance of one or more T1DM-specific autoantibodies in the circulation (seroconversion), are of particular relevance [71,72,73,76,77,78,79,80,82]. Longitudinal studies in large series of children are also of paramount importance as they can prove that microbiome abnormalities precede seroconversion and/or clinical T1DM onset [71,73,76,77,78,80,82].

However, establishing a causal relationship between microbiome alterations and T1DM in humans is very challenging because of the complexity of the microbiota immune system cross talk. It is also difficult to identify microbiota abnormalities consistently associated with diabetes, because seroconversion occurs in early life when the microbiota is unstable and it undergoes substantial changes over time [25,26]. Furthermore, factors as delivery mode, diet, geographical location, and lifestyle dramatically affect the microbiota and, besides being potential causes of dysbiosis, they can also act as relevant confounders [27,29,32]. Although causality between dysbiosis and T1DM has not yet been proven in humans, as there are no placebo-controlled trials showing that long-term changes in the intestinal microbiota modify the risk of T1DM, there are data suggesting a role of the gut microbiota in the development of human T1DM. 

Children diagnosed with T1DM have reduced gut microbiota diversity in fecal samples. A lower microbial diversity was also shown in children with at least two positive disease-associated autoantibodies compared to autoantibody-negative children. Moreover, longitudinal studies in children at risk of T1DM have shown that the decrease in microbial diversity occurs after seroconversion, but prior to T1DM onset [71,72,73]. 

Several studies on the microbiota composition in T1DM reported an increase in Bacteroidetes (Gram negative) and a decrease in Firmicutes (Gram positive) [72,73,74,75]. The prevalent species from the Firmicutes phylum produce protective SCFAs, including butyrate [83], suggesting that the reduction in Firmicutes seen in T1DM may be deleterious because of diminished butyrate production. On the other hand, the Bacteroidetes phylum is dominated by Bacteroides and Prevotella [19]. In T1DM, the quantity of Bacteroides is significantly higher, whereas that of Prevotella is significantly lower than in healthy controls and this is of relevance, as Bacteroides have been associated with gastrointestinal inflammation and increased intestinal permeability, while Prevotella appear protective. In a cross-sectional study an increased abundance of Bacteroides characterized the microbiota of children who seroconverted compared with non-converters [72]. Similarly, a study performed in 76 Finnish children found increased Bacteroides dorei and Bacteroides vulgatus species in seroconverted T1DM patients. Of interest, longitudinal data from this study showed that the increase in Bacteroides dorei peaked at 7.6 months of age in autoantibody-positive children and preceded the appearance of the first anti-islet autoantibodies, suggesting that early dysbiosis may predict T1DM in genetically predisposal subjects [76]. The increased abundance of Bacteroides species in Finnish and Estonian compared to Russian children has been even proposed as a possible explanation for their higher incidence of T1DM [84]. Bifidobacterium species were also reduced in autoantibody-positive compared to autoantibodies negative children [72] and this may be of functional relevance as Bifidobacteria contribute to the production of butyrate and inhibit bacterial translocation through the gut barrier. 

However, recent metagenomic studies suggest that changes in microbiome function are more relevant and consistently associated with the risk of seroconversion and/or T1DM onset than changes in specific taxa. The Environmental Determinants of Diabetes in the Young (TEDDY) study did not find important taxonomic differences in the gut microbiome of children who seroconverted or developed T1DM. However, metagenomic analysis revealed that the microbiome of these children contained significantly lower numbers of genes involved in the production of SCFAs [77]. This finding is in line with a previous study by de Goffau et al., reporting that β cell autoimmunity prior to T1DM onset is associated with a reduction in butyrate-producing species [72]. Dietary factors may induce changes in the composition of the gut microbiome, leading to both reduced butyrate production and enhanced risk of autoantibodies associated to the development of T1DM [78]. A recent metaproteomic analysis has also shown that microbial taxa associated with host proteins involved in maintaining function of the mucous barrier and microvilli adhesion were depleted in patients with new-onset T1DM [79]. Moreover, intestinal permeability was increased in children at risk of developing T1DM and correlated with microbiota alterations [85,86]. These findings support the notion that increased gut permeability is important in linking the microbiome to T1DM in humans as previously demonstrated in animal model of autoimmune diabetes. 

Adding a further layer of complexity to the field, a recent study has shown that *HLA* can influence the composition of the human gut microbiome [80]. Indeed, genetic risk for developing T1DM autoimmunity was associated with distinct changes in the gut microbiome. Therefore, in T1DM genetic susceptibility can not only synergically interact with, but also contribute to shape the microbiome [80]. This finding may also be relevant for the design of future clinical studies as recruitment of children at high genetic risk for T1DM is less desirable than we previously thought because it can mask the effect of *HLA* on the microbiome.

Overall data in humans are more conflicting than in experimental animals. Most of the available data in humans come from underpowered case-control studies and this may partially explain inconsistencies. Moreover, human studies mainly prove associations between dysbiosis and T1DM rather than a causal relationship and they explored taxonomic rather than functional changes in the microbiota. New approaches such as metagenomics and metaproteomics applied to large cohort of patients and intervention studies may provide better insights on this topic in the future and move this area of research from a description of abnormalities to translational applications.

## 6. Future perspective

Research on the microbiome in T1DM has expanded dramatically in recent years; however, a causal relationship between dysbiosis and the risk of T1DM has not yet been established in humans. Even though intervention studies in susceptible animals have provided evidence that strategies ameliorating/reversing dysbiosis reduce the incidence of T1DM, this evidence is still lacking in humans. However, tailored changes in the intestinal microbiome may represent a novel therapeutic approach for the primary prevention of T1DM and various strategies have been proposed to reshape the gut microbiota in children at high risk of T1DM. Infants delivered by C-section can be exposed to maternal vaginal fluids at birth to make their microbiota composition comparable to that of vaginally born babies [87]. Supplementation with LMP dietary fiber may be beneficial in humans as recently proven in mice. Prebiotics, such as inulin, lactulose, and in particular human milk oligosaccharides, can promote the growth of Bifidobacteria and a recent pilot study showed that treatment for 3 months with the prebiotic oligofructose-enriched inulin in children with T1DM significantly increased C-peptide levels [81]. Clinical trials are currently testing the efficacy of supplementation with probiotics in human T1DM [88]. Notably, a recent study demonstrated that the administration of probiotics in the first month of life was associated with a 60% reduction in the autoimmunity risk in children with the high-risk *HLA-DR3/4* genotype [82]. Moreover, a clinical trial with the probiotics Lactobacillus rhamnosus GG and Bifidobacterium lactis Bb12L is currently ongoing in children with new-onset T1DM [89]. In the era of precision medicine, personalized diets, tailored in accordance with both the host’s genetic background and individual microbiome may hopefully correct dysbiosis and reduce the risk of developing T1DM.

## Figures and Tables

**Table 1:** Type 1 Diabetes and the Microbiota: Human Studies.

Study Design	Study Sample	Main Findings	Ref.
Prospective	In total, 33 genetically predisposed infants	Microbial diversity in T1DM progressors in the time window between seroconversion and T1DM onset and inflammation-favoring organisms	[71]
Case Control	In total, 18 Ab (+) vs. 18 Ab (-) children matched for *HLA*	Ab-positive children: microbial diversity, *Bacteroidetes* (*Bacteroides* and *Prevotella)*, Bifidobacterium species, short-chain fatty acid (SCFA)-producing bacteria	[72]
Case Control (longitudinal data)	In total, four *HLA*-matched case (Ab+) –control (Ab-) pairs (three time points)	*Bacteroidetes* (Bacteroidales) and *Firmicutes* (*Clostriales*) in cases vs. controls at all time points. Children progressing T1DM: microbial diversity	[73]
Case Control	In total, 28 newly diagnosed type 1 diabetes (T1DM) (average duration 4.8 years) vs. 27 age-matched control children	In children < 3 years: *Bacteroidetes* in cases and *Clostridium clusters IV* and *XIVa* in controls	[74]
Case Control	In total, 16 T1DM vs. 16 healthy children	Cases: Bacteroidetes, Firmicutes and Actinobacteria	[75]
Case Control (longitudinal data)	In total, 29 Ab (+) cases vs. 47 Ab (-) healthy controls	*B. dorei* and *B. vulgatus* in cases prior to seroconversion	[76]
Prospective	In total, 783 genetically predisposed children	SCFA-producing bacteria genes in children who seroconverted or developed T1DM	[77]
Prospective	In total, 19 Ab (+) and 21 Ab (-) children	*Bacteroides Akkermansia* SCFA-producing bacteria genes. A functional association between diet (early introduction of non-milk diet), gut microbiome (*Bacteroides*), metagenomic changes (genes for the production of butyrate) and development of islet Ab	[78]
Case Control	In total, 33 recent-onset T1DM, 17 Ab (+), 29 Ab (-), and 22 healthy subjects	T1DM: microbial taxa associated with host proteins involved in maintaining the function of the mucous barrier, microvilli adhesion, and exocrine pancreas	[79]
Cohort Study	In total, 403 children (age = 1 year)	Genetic risk for developing T1DM autoimmunity is associated with distinct changes in the gut microbiome	[80]
RCT	T1DM children (8–17 years) randomized to prebiotic oligofructose-enriched inulin (*n* = 17) or placebo (*n* = 21) for 12 weeks	At 3 months, C-peptide was significantly higher in the group that received prebiotics	[81]
Prospective Cohort	In total, 7473 children (4–10 years)	Early probiotic supplementation (at the age of 0–27 days) associated with a decreased risk of islet autoimmunity in children with the *HLADR3/4* genotype	[82]

T1DM: type 1 diabetes, RCT: randomized control trial, HLA: human leukocyte antigen, Ab (+): islet auto-antibodies positive, and Ab (-): islet auto-antibodies negative.
